# Ernest Beutler (1928-2008)

**Published:** 2009

**Authors:** Kanjaksha Ghosh, Ajit C. Gorakshakar

**Affiliations:** National Institute of Immunohaematology, 13^th^ Floor, New Multistoried Bldg, K.E.M. Hospital Campus, Parel, Mumbai-400 012, India

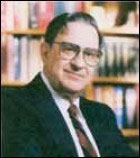



The world of hematology and genetics lost an academia giant, Dr. Ernest Beutler, on October 5, 2008 at the age of 80. He studied blood diseases, genetics, iron metabolism, and Gaucher's disease to name a few.

Dr Beutler, over five decades during the 20^th^ century, made notable contributions to the understanding of a wide range of human diseases like malaria, hemochromatosis, iron overload, leukemia, etc. His publications on these subjects include more than 800 original articles, numerous chapters, monographs, and books.

The world especially remembers him for his seminal contribution in understanding drug (Primaquine)-induced hemolysis in malarial infection. He, through his painstaking work, linked this hemolysis to G6PD deficiency and spent a good part of his life in studying G6PD deficiency in particular and red cell enzymopathies in general. He devised several simple spot tests to detect G6PD deficiency and pyruvate kinase deficiency by field workers who are not experts in red cell enzyme studies. His manual on red cell enzymes[1] is like a Bible to many of us who study red cell enzymes.

In 1980, he evaluated a new drug, 2 chlorodeoxy adenosine (2CdA), which was found to be highly effective in treating hairy cell leukemia.[2] This was an important practical contribution to cancer research.

He was concerned about the high cost of treatment for Gaucher's disease using enzyme replacement therapy (Ceredase). Hence, he tried to solve this problem using substrate reduction therapy with some success.[3] His group cloned the gene responsible for this disease.

Dr Beutler also worked on Tay-Sachs disease, galactosemia, sickle cell anemia, and other hemolytic anemias. He identified a genetic mutation causing hemochromatosis especially in Africans. He started a marrow transplantation program and published the first paper supporting the use of allogeneic marrow transplantation as primary treatment of acute leukemia.[4]

Dr Beutler was selected to receive numerous awards like a MERIT Award from the NIH, the Gairdner Foundation Award (1975), the James Blundell Award of the British Transfusion Society (1985), the City of Medicine Award (1994), and the Philip Levine Award (2000) to name a few. He has served on the editorial board of 18 journals of hematology, molecular medicine, and nutrition sciences. He founded the journal “Blood Cells, Molecules and Diseases.”

The Editor of this journal had the opportunity to interact with Dr. Beutler in 2005 during a meeting[5] on G6PD deficiency and drug trials. He was very excited to learn that my institute, i.e. the National Institute of Immunohaematology, is continuing work on red cell enzymopathies, his first love. When I requested for some help in this area, he felt sad and told me that he had moved away from red cell enzymes as the chairman of the department of molecular and experimental medicine at Scripps Research Institute, (a post he relinquished just a month before his death) but would like to help us in any other way he could. I, as the editor of the Indian Journal of Human Genetics, requested him to submit a review article to this journal. He readily agreed and submitted the article.[6]

He also insisted that we should not give up red cell enzyme studies as there are very few centers in the world that are pursuing this area of research.

I was impressed by his knowledge, erudition, and gentleness. His work is like a beacon that will continue to inspire generations of red cell enzymologists.

May his soul rest in peace.
